# Malgaigne's Operative Surgery

**Published:** 1847-01

**Authors:** 


### ART. X.

*Malgaigne's Operative Surgery.* Translated from the French by F. BRITTAN, A.B., M.D., &c. &c.—*London,* 1846. 12mo, pp. 586.

THE Manual of Operative Surgery by Malgaigne is so well known to the profession as one of the most comprehensive works on the subject of which it treats, and enjoys so extensively diffused a reputation amongst the surgeons of the Continent,—having gone through four editions in the original, and been translated into five continental languages,—that it will be unnecessary for us to do more than to name to such of our surgical readers as are unacquainted with them the principal merits of the work. We are surprised that it has not sooner made its appearance in an English dress.

The chief value of M. Malgaigne's work consists, first, in its very comprehensive and compact character, embracing as it does in a small compass the whole range of operative surgery; and, secondly, in the extreme clearness and precision with which details, whether of surgical anatomy or of operative manipulation, are described. In these respects—the especial desiderata to be met with in a *manual*—it stands unrivalled by any similar treatise on the same subject in any language.

The work is divided into three parts. In the first, the author treats of what he terms the "Elements of operations," such as the management of the knife, of cauteries, and of ligatures or sutures; with some short remarks on the plans that have been suggested for lessening pain during operations. In the second part, general operations, from bloodletting and plugging teeth to the ligature of arteries and the resection of joints, are fully and clearly described. In the third part, we have a very complete account of special operations.

In describing an operation, the author first gives, in a very concise manner, the surgical anatomy of the part concerned; he then describes minutely, step by step, the ordinary method of operating; this is followed by an account of the principal modifications that have been, or are, adopted by different operators; and he concludes with a comparison between the different methods, a general appreciation of the operation as a whole, and with cautions as to the dangers that may occur during its performance, or by which it may be followed.

The following extract will convey to our readers a good idea of the plan adopted in describing an operation, as well as of the style of the translation.

"PHYMOSIS. The surgical treatment of phymosis comprises three methods: incision, excision, and circumcision.

"1. INCISION. *Ordinary proceeding.* The patient is seated on a chair, with his back applied against a wall so that he cannot start back; or laid on the right side of his bed. The surgeon pinches up the right side of the opening of the prepuce, and draws it a little forwards; he then insinuates a director between the glans and prepuce in the median line, and on the superior surface, as far as the cul-de-sac of the mucous membrane. An assistant supports the penis and draws back the skin, so that the incision shall not go too far. The surgeon himself holds the director in his left hand, and with his right passes a thin, narrow-bladed, sharp-pointed, straight bistoury along its groove. When he reaches the end of the director he depresses his right hand, and brings out the point of his knife through the skin; and then sharply drawing it towards himself, he divides the prepuce from before backwards in one cut.

"The skin is usually divided further than the mucous membrane, and the latter is subject to form a small cul-de-sac beyond the incision: it should be divided with scissors.

"Some surgeons advise placing a small ball of wax on the point of a bistoury, and introducing it flat without a director between the glans and prepuce; and then, turning its edge up, finishing the incision as usual. For this the blade must be very narrow, or it will wound the glans or prepuce in its passage. We prefer the ordinary proceeding.

"*Proceeding of Cloquet.* It only differs from the former in being performed on the inferior part of the prepuce at one side of the frænum. It is described by Celsus.

"*Proceeding of Cullerier.* He divides the mucous membrane of the prepuce, only beginning at its edge as if to free the opening.

"*Proceeding of Carter.* This consists in three superficial incisions on the free edge of the prepuce, and on its dorsal and lateral surfaces.

"M. Malapert modified this proceeding by making two incisions at the prepuce, and the third at the frænum. These incisions, made at equal intervals, should be of proportionate extent to the constriction of the orifice—from a quarter to half an inch but never more. . . . . . .

"*Appreciation.* Incision by the proceedings of Cullerier or Carter may well be used in cases where an accidental swelling has constricted a prepuce that is otherwise large enough. But in natural phymosis, or when the edge of the prepuce is ulcerated or indurated, more powerful means must be adopted. Incision by the ordinary means leaves two long loose flaps hanging about, more or less tumefied; by that of Cloquet, a long, thick, deformed, flap: excision, usually by the proceeding of Lisfranc, is then indispensable. When all the free edge of the prepuce should be sacrificed, recourse must be had to circumcision, for which the proceeding of Ricord seems the easiest.

"Whatever proceeding is preferred you should bear in mind that the prepuce is not united to the glans by a circular commissure; but that this commissure follows the corona glandis; and that as it is oblique from above downwards, and from before backwards, in this direction the incision should be made.

"Whatever proceeding is adopted, you may apply to it a modification proposed by Mr. Hawkins, of St. George's Hospital, London. It consists in reuniting the skin and mucous membrane by means of four or five points of suture. In this way union by the first intention is often obtained; whilst, by the old method, there was often a nasty ulceration of the wound for a long time." (pp. 471-5.)

In giving an English version of Malgaigne's Manual, Mr. Brittan has filled a hiatus that existed in the surgical literature of this country. With the exception of Hargrave's Operative Surgery, which,—in the clearness of its details, and the comprehensiveness of the information it contains, resembles Malgaigne's work more nearly than any other in our language, but which is now out of date, and we believe out of print,—English surgeons do not possess a complete treatise on the operations of surgery. The works of Liston and of Fergusson, excellent though they be, are incomplete, in not containing the details of many important operations, and rather represent the peculiar practice of their respective authors, or describe that which is most generally adopted in this country, than give a general view of the whole range of operative surgery. To these works, therefore, the volume before us will be a useful adjunct; containing as it does a vast deal of information, especially relating to the practice of continental surgeons, that we shall seek for in vain in any other treatise in our language.

Mr. Brittan has executed the task he had imposed upon himself in a very judicious and praiseworthy manner. The translation is extremely readable, and, although a close and faithful version of the original, is rendered in an agreeable and easy style, being totally devoid of Gallicisms. The value of the original, has likewise been much increased by the addition by Mr. Brittan of notes containing the details of a few operations omitted by the author, and of some that have been introduced into practice since the publication of the last French edition in 1842; of this kind are those of Fergusson and Dieffenbach on Staphyloraphy, of Clay and others on Ovariotomy, &c., and giving the opinions of some of the more celebrated English surgeons where they are at variance with the doctrines advocated by Malgaigne. The illustration of the work by some very well-executed woodcuts, representing the more important surgical regions, has added much to its utility, and we can strongly recommend it both to practitioners and students, not only as a safe guide in the dissecting-room or operating theatre, but also as a concise work of reference for all that relates to operative surgery.

---

